# A Four-Year Analysis of #OrthoTwitter Use During Match Week: Can X Be a Potential Equalizer for Students Left Unmatched?

**DOI:** 10.7759/cureus.115033

**Published:** 2026-08-23

**Authors:** Desiree E Ojo, Davis Hedbany, Brook Willett, Brandon D Rust, Geoffrey Deckard, Omar Guerrero, Jalen Warren, Michael Megafu, Morgan Turnow, Jaime Bellamy

**Affiliations:** 1 Orthopedic Surgery, Inspira Health, Philadelphia, USA; 2 Orthopedic Surgery, State University of New York Upstate, Syracuse, USA; 3 Orthopedic Surgery, University of the Incarnate Word, San Antonio, USA; 4 Orthopedic Surgery, Naval Medical Center Portsmouth, Davie, USA; 5 Radiology, Texas Tech University Health Sciences Center, Lubbock, USA; 6 Orthopedic Surgery, Cleveland Clinic, Cleveland, USA; 7 Orthopedic Surgery, University of Connecticut, Hartford, USA; 8 Orthopedics, OhioHealth Doctors Hospital, Columbus, USA; 9 Arthroplasty and Trauma, University of Missouri–Kansas City, Kansas City, USA

**Keywords:** graduate medical education, medical education, orthopedic surgery, orthotwitter, osteopathic medicine, social media

## Abstract

Background

The orthopedic surgery match has changed. Since the COVID-19 pandemic, orthopedic surgery candidates and residency programs have used #Orthotwitter to promote, advocate, and educate their followers. This study aimed to evaluate changes in the use of #OrthoTwitter and #OrthoMatch during Match Week over a four-year period, including trends in author type, post content, institutional affiliation, engagement, and posting activity, with particular attention to posts involving unmatched applicants.

Methodology

The top tweets containing the hashtags #Orthotwitter and/or #OrthoMatch during the 2021, 2022, 2023, and 2024 Match Week were reviewed and collected. Tweets between the years 2021, 2022, 2023, and 2024 were compared using the chi-square test. Differences in engagement between author type and content were analyzed using the Kruskal-Wallis H test.

Results

Of the 370 tweets in total, 25.6% (n = 92) were from 2024, 24.5% (n = 93) from 2023, 25% (n = 95) from 2022, and 23.7% (n = 90) from 2021. Over the course of four years, medical students (47.7%, n = 179) tweeted the most during #Orthotwitter Match week, followed by attendings (26.6%, n = 100), and third-party organizations (9.6%, n = 36).

Conclusions

#Orthotwitter over the course of the past four years has shifted from a focus on the thoughts of attendings and third-party organizations to medical students. Students are likely to share a negative match result, and third-party organizations and attendings are likely to tweet resources for unmatched orthopedic surgery candidates. As the #Orthomatch continues to evolve, we anticipate that Twitter can become a potential equalizing platform for students who are left unmatched on Match Day.

## Introduction

Social media offers a global platform for users to create, share, receive, and connect with others effortlessly [1]. In the United States alone, 80% of the population utilizes internet-based sites for social networking [2]. Healthcare professionals use these platforms to engage students, share scientific information, and interact with other practitioners and the general public [3]. Following the COVID-19 pandemic, there has been a surge in activity across many of these platforms [1,4].

Among the most popular healthcare social media platforms, X (formerly Twitter, rebranded in July 2023; referred to hereafter as X) stands out with over 400 million active monthly users [5,6]. The app uses a brief character limit and hashtags (i.e., the symbol “#” followed by a word) to classify and label information, facilitating ease of dissemination [6]. “#OrthoTwitter” is a standard hashtag for a wide range of information related to orthopedic surgery, encompassing patient-related content, promotional news, and personal tweets [7]. For orthopedic surgery residency applicants, “#OrthoTwitter” proved invaluable during the COVID-19 pandemic, enabling students to showcase their abilities, identities, and interests to prospective programs at a time when away rotations were discouraged [8]. Today, “#OrthoTwitter” provides a nontraditional avenue for education, mentorship, and networking within the orthopedic community [4]. Prospective applicants to orthopedic residency programs continually rely on this platform to navigate the period leading up to and following Match Week [8]. This study aims to analyze the nature of tweets and interactions within #OrthoTwitter, with specific analysis during the orthopedic surgery residency match period from 2021 to 2024.

This article was previously presented as a meeting abstract by Brook Willett in San Antonio at the 2025 Mid-America Orthopaedic Association meeting on April 12th, 2025.

## Materials and methods

X was utilized to identify content relevant to orthopedic residency discourse. The top tweets containing the hashtags “#OrthoTwitter” and/or “OrthoMatch” from 2021 to 2024 were reviewed. The X platform uses a sorting system to categorize which 100 tweets rise to the top based on engagement, relevance, and user interactions. The specific periods of tweet collection were from Monday to Friday in March of the 2021, 2022, 2023, and 2024 National Resident Matching Program Match Weeks. Original tweets were included, and replies and quote tweets were excluded from the analysis.

The following information was manually collected for each tweet: author type, content, author statistics, activity before the match, activity after the match, and institution association. Author types were defined as medical student, resident or fellow, Program Director/Assistant Program Director (PD/APD), residency program, attending physician, third-party organization, and others. Content was defined as congratulatory messages, advice for unmatched applicants, announcements of positive match results, announcements of not matching, acknowledgment of underrepresented minorities (URMs) in orthopedics, information about research years for unmatched applicants, and other. Author statistics were defined as Doctor of Osteopathic Medicine (DO), Doctor of Medicine (MD), or neither. Activity before and after the Match was defined as active or inactive. Institutional association was defined as allopathic, osteopathic, international medical graduate (IMG), private practice/group, and other. Data regarding author characteristics were collected from the X biography of each user.

Tweets were manually reviewed on an individual basis (DO, OG, JW, BW, GD). Any discrepancies in data collection were resolved through group discussion and consensus. Tweets from 2021 to 2024 were pooled and analyzed for overall trends, descriptive characteristics, and engagement. Tweet engagement was assessed using two metrics: the number of likes and the combined total of retweets and replies per tweet. The differences in engagement by author type and content were examined using the Kruskal-Wallis H test. A post-hoc analysis was performed using the Bonferroni adjustment. To account for the number of followers of each account, a sensitivity analysis was performed by dividing the engagement measures by the number of followers for each account.

From 2021 to 2024, tweet characteristics, including author type, content type, author credential statistics, activity before and after the match, and institution association, were compared using chi-square tests. All statistical analyses were two-sided, with a significance threshold of p < 0.05. Analyses were performed using SPSS Statistics version 29.0.2.0 (IBM Corp., Armonk, NY, USA).

## Results

Overall, 370 individual tweets were reviewed and analyzed. The most common authors were medical students, who authored 179 (47.7%) tweets. The overview and comparison of all tweets are presented in Table 1. The most common type was an announcement of a positive match result, with 146 (39.3%) tweets.

**Table 1:** Overview and comparison of tweets from 2021 to 2024 on OrthoTwitter. PD = Program Director; APD = Assistant Program Director; URMs = underrepresented minorities; MD = Doctor of Medicine; DO = Doctor of Osteopathic Medicine; IMG = international medical graduate

Year	2021	2022	2023	2024
Total tweets (n) (sum = 370)	90	95	93	92
Author type				
Medical student, n (%)	36 (40.0%)	53 (55.8%)	56 (60.2%)	34 (36.9%)
Resident/Fellow, n (%)	2 (2.2%)	4 (4.2%)	7 (7.5%)	4 (4.3%)
PD/APD, n (%)	5 (5.6%)	5 (5.3%)	2 (2.2%)	3 (3.2%)
Residency program, n (%)	4 (4.4%)	0 (0.0%)	0 (0.0%)	3 (3.2%)
Attending, n (%)	33 (36.7%)	22 (23.2%)	18 (19.4%)	27 (29.3%)
Third-party organization, n (%)	6 (6.7%)	8 (8.4%)	7 (7.5%)	15 (16.3%)
Other, n (%)	4 (4.4%)	3 (3.2%)	3 (3.2%)	6 (6.5%)
Content, n (%)				
Congratulatory message, n (%)	26 (28.9%)	11 (11.6%)	3 (3.2%)	15 (16.3%)
Advice for unmatched applicants, n (%)	2 (2.2%)	2 (2.1%)	3 (3.2%)	0 (0.0%)
Announcement of positive match result, n (%)	27 (30.0%)	44 (46.3%)	45 (48.4%)	30 (32.6%)
Announcement of not matching, n (%)	1 (1.1%)	10 (10.5%)	6 (6.5%)	7 (7.6%)
Acknowledgement of URMs in Ortho, n (%)	4 (4.4%)	1 (1.1%)	0 (0.0%)	0 (0.0%)
Applicants, n (%)	0 (0.0%)	6 (6.3%)	7 (7.5%)	4 (4.3%)
Other, n (%)	30 (33.3%)	21 (22.1%)	29 (31.2%)	41 (44.5%)
Author statistics				
MD, n (%)	89 (98.9%)	81 (85.3%)	72 (77.4%)	45 (48.9%)
DO, n (%)	1 (1.1%)	3 (3.2%)	13 (14.0%)	18 (19.5%)
Neither, n (%)	0 (0.0%)	11 (11.6%)	8 (8.6%)	34 (36.9%)
Activity before match				
Active, n (%)	89 (98.9%)	87 (91.6%)	92 (98.9%)	91 (98.9%)
Inactive, n (%)	1 (1.1%)	8 (8.4%)	1 (1.1%)	1 (1.1%)
Activity after match				
Active, n (%)	89 (98.9%)	85 (89.5%)	88 (94.6%)	90 (97.8%)
Inactive, n (%)	1 (1.1%)	10 (10.5%)	5 (5.4%)	2 (2.2%)
Institution association				
Allopathic, n (%)	71 (78.9%)	67 (70.5%)	60 (64.5%)	47 (51%)
Osteopathic, n (%)	1 (1.1%)	4 (4.2%)	11 (11.8%)	16 (17.3%)
IMG, n (%)	9 (10.0%)	3 (3.2%)	7 (7.5%)	2 (2.1%)
Private Practice/Group, n (%)	0 (0.0%)	7 (7.4%)	6 (6.5%)	10 (10.8%)
Other, n (%)	9 (10.0%)	14 (14.7%)	9 (9.7%)	22 (23.9%)

Analysis of X engagement

Data regarding X engagement, including median likes, retweets/replies, and the number of followers, are presented in Table 2. All p-values and sensitivity analyses for group comparisons are available in Tables 3-6.

**Table 2:** Engagement and followers by author type and content category. PD = Program Director; APD = Assistant Program Director; URMs = underrepresented minorities; RY = resident year; IQR = interquartile range

	Median likes (IQR)	Median retweets + comments (IQR)	Median followers (IQR)
Author type			
Medical student	515 (204–1,100)	39 (15.5–84.5)	504 (278–929.5)
Resident/Fellow	64 (22–73)	10 (5–40)	1,244 (465–1,868)
PD/APD	40 (22.5–77.5)	3 (2.75–5.25)	2,534.5 (2,226.25–4,178.5)
Residency program	13 (9–17)	4 (2.5–5.5)	1,732 (821–2,550)
Attending	36.5 (23.5–64)	5.5 (2–15)	3,599 (1,687–9,159.5)
Third-party organization	15 (9.5–26.25)	8 (3–13.25)	3,363.5 (1,730–15,050)
Other	31.5 (18.5–65)	9.5 (6.75–18.75)	1,299 (847.25–2,230)
Content type			
Congratulatory message	45 (25–73.5)	3 (2–8)	2,160 (840.5–3,449)
Advice for unmatched applicants	50 (37.5–79)	9 (4–28.5)	2,113 (1,257.5–3,850)
Announcement of positive match result	654 (298.75–1,200)	41.5 (15.25–88.75)	471 (266.25–876)
Announcement of not matching	333.5 (124.75–1,100)	59 (29.75–90)	502 (293.25–1,052.75)
URM applicant recognition	62 (51–74)	4 (3–8)	1,300 (1,000–1,500)
Info about RY for unmatched applicants	35 (26–62)	16 (13–28)	1,798 (833–4,938)
Other	25 (13–50)	8 (3–15)	3,400 (1,716–10,900)

**Table 3:** Sensitivity analysis of tweet engagement after adjusting for number of account followers (author type). PD = Program Director; APD = Assistant Program Director; IQR = interquartile range

	Likes per follower (IQR)	Retweets + comments per follower (IQR)
Medical student	1.2693 (0.4933–3.0360)	0.0848 (0.0342–0.2067)
Resident/Fellow	0.0430 (0.0104–0.2857)	0.0055 (0.0019–0.0735)
PD/APD	0.0100 (0.0055–0.0289)	0.0010 (0.0006–0.0024)
Residency program	0.0058 (0.0048–0.0158)	0.0012 (0.0011–0.0071)
Attending	0.0108 (0.0044–0.0278)	0.0014 (0.0005–0.0057)
Third-party organization	0.0040 (0.0012–0.0104)	0.0011 (0.0003–0.0034)
Other	0.0251 (0.0120–0.1262)	0.0080 (0.0036–0.0398)

**Table 4:** Sensitivity analysis of tweet engagement after adjusting for number of account followers (content type). URMs = underrepresented minorities; RY = resident year; IQR = interquartile range

Content type	Likes per follower (IQR)	Retweets + comments per follower (IQR)
Congratulatory message	0.0159 (0.0085–0.061)	0.0013 (5e-04–0.0046)
Advice for unmatched applicants	0.0183 (0.0121–0.0661)	0.0043 (9e-04–0.0327)
Announcement of positive match result	1.6852 (0.7561–3.3134)	0.096 (0.0426–0.2031)
Announcement of not matching	0.7384 (0.2904–4.1151)	0.1426 (0.0425–0.2957)
Acknowledgement of URMs in Ortho	0.0477 (0.0327–0.0532)	0.0027 (0.0023–0.0031)
Info about RY for unmatched applicants	0.0195 (0.0079–0.0493)	0.0073 (0.0032–0.0256)
Other	0.0071 (0.0024–0.0186)	0.0015 (5e-04–0.0057)

**Table 5:** P-values for differences in engagement by author type. PD = Program Director; APD = Assistant Program Director

Author type	Medical student	Resident/Fellow	PD/APD	Residency program	Attending	Third-party organization	Other
Likes							
Resident/Fellow	<0.001	-	-	-	-	-	-
PD/APD	<0.001	1	-	-	-	-	-
Residency Program	<0.001	0.365	1	-	-	-	-
Attending	<0.001	1	1	1	-	-	-
Third-party organization	<0.001	0.114	1	1	0.22	-	-
Other	<0.001	1	1	1	1	0.837	-
Retweets/Comments							
Resident/Fellow	0.0606	-	-	-	-	-	-
PD/APD	<0.001	0.306	-	-	-	-	-
Residency Program	<0.001	1	<0.001	-	-	-	-
Attending	<0.001	1	1	1	-	-	-
Third-party organization	<0.001	<0.001	<0.001	<0.001	<0.001	-	-
Other	0.0286	1	0.636	1	1	1	-
Likes per follower							
Resident/Fellow	0.00205	-	-	-	-	-	-
PD/APD	<0.001	1	-	-	-	-	-
Residency Program	<0.001	1	<0.001	-	-	-	-
Attending	<0.001	0.505	1	1	-	-	-
Third-party organization	<0.001	<0.001	<0.001	<0.001	<0.001	-	-
Other	0.00119	1	1	1	1	0.0879	-
Retweets/Comments per follower							
Resident/Fellow	0.016	-	-	-	-	-	-
PD/APD	<0.001	0.325	-	-	-	-	-
Residency Program	0.00181	1	<0.001	-	-	-	-
Attending	<0.001	0.244	1	1	-	-	-
Third-party organization	<0.001	<0.001	<0.001	<0.001	<0.001	-	-
Other	0.0358	1	0.27	1	0.2	0.0628	-

**Table 6:** P-values for differences in engagement by content type. URMs = underrepresented minorities; RY = resident year

Content type	Congratulatory message	Advice for unmatched applicants	Announcement of positive match result	Announcement of not matching	Acknowledgement of URMs in Ortho	Info about RY for unmatched applicants	Other
Likes							
Advice for unmatched applicants	1	-	-	-	-	-	-
Announcement of positive match result	<0.001	<0.001	-	-	-	-	-
Announcement of not matching	<0.001	<0.001	1	-	-	-	-
Acknowledgement of URMs in Ortho	1	1	0.314	1	-	-	-
Info about RY for unmatched applicants	1	<0.001	<0.001	<0.001	<0.001	-	-
Other	0.88	1	<0.001	<0.001	1	1	-
Retweets/Comments							
Advice for unmatched applicants	1	-	-	-	-	-	-
Announcement of positive match result	<0.001	<0.001	-	-	-	-	-
Announcement of not matching	<0.001	<0.001	1	-	-	-	-
Acknowledgement of URMs in Ortho	1	1	0.0422	0.0114	-	-	-
Info about RY for unmatched applicants	0.00231	<0.001	<0.001	<0.001	<0.001	-	-
Other	0.313	1	<0.001	<0.001	1	0.187	-
Likes per follower							
Advice for unmatched applicants	1	-	-	-	-	-	-
Announcement of positive match result	<0.001	<0.001	-	-	-	-	-
Announcement of not matching	<0.001	<0.001	1	-	-	-	-
Acknowledgement of URMs in Ortho	1	1	0.412	1	-	-	-
Info about RY for unmatched applicants	1	<0.001	<0.001	<0.001	<0.001	-	-
Other	0.194	1	<0.001	<0.001	1	1	-
Retweets/Comments per follower							
Advice for unmatched applicants	1	-	-	-	-	-	-
Announcement of positive match result	<0.001	<0.001	-	-	-	-	-
Announcement of not matching	<0.001	<0.001	1	-	-	-	-
Acknowledgement of URMs in Ortho	1	1	0.105	0.0651	-	-	-
Info about RY for unmatched applicants	1	<0.001	<0.001	<0.001	<0.001	-	-
Other	1	1	<0.001	<0.001	1	1	-

Tweets authored by medical students received the most likes (median = 515, interquartile range (IQR) 204-1,100) and retweets/replies (median = 39, IQR = 15.5-84.5). Residents and fellows followed closely in terms of engagement (likes: median = 64, IQR = 22-73; retweets/replies: median = 10, IQR = 5-40). The number of likes and retweets/replies received by tweets from medical students was significantly greater than those authored by PD/APDs, residency programs, orthopedic attendings, and third-party organizations (p < 0.001). Once we controlled for the number of followers, medical students received significantly more likes and retweets/replies than residents/fellows (p = 0.002 and p = 0.016, respectively), PD/APDs (p < 0.001 for both), residency programs (p < 0.001 and p = 0.002, respectively), orthopedic attendings (p < 0.001 for both), and third-party organizations (p < 0.001 for both).

Tweets focusing on the announcement of positive match results received the most likes (median = 654, IQR = 298.75-1,200) and retweets/replies, followed by personal announcements of not matching (likes: median = 333.5, IQR = 124.75-1100). Engagement with these content types was significantly greater than tweets providing advice for unmatched applicants and information about research years for unmatched applicants (p < 0.0001). There was no significant difference in the number of likes between tweets about positive match (p = 0.314) results or not matching (p = 1).

Comparison between 2021 and 2024

From 2021 (90 tweets) to 2024 (92 tweets), there was a 2.2% increase in the number of tweets. There was no significant difference in the number of tweets from any specific author type between 2021 and 2024. However, there was a significant decrease in tweets with congratulatory messages (28.9% vs. 15.5%, p = 0.041). Conversely, there was a significant increase in tweets acknowledging not matching (1.1% vs. 7.6%, p = 0.035). Additionally, the number of tweets from DO physicians increased significantly (1.1% vs. 18.6%, p < 0.001), with a corresponding decrease in tweets from MD physicians (98.9% vs. 46.4%, p < 0.001). Institutional association analysis showed a significant rise in tweets from osteopathic institutions (1.1% vs. 16.5%, p = 0.001) and private practice groups (0.0% vs. 10.3%, p = 0.005), and a decrease in tweets from allopathic institutions (78.9% vs. 48.5%, p < 0.001) and IMG institutions (10.0% vs. 2.1%, p = 0.046).

## Discussion

The COVID-19 pandemic has revolutionized the way orthopedic surgery-interested medical students network, stay informed, and advocate for themselves as future residency applicants [9-12]. With the pivot to virtual interviews, open houses, and meet-and-greets, students have turned to social media to network, seek mentorship and research opportunities, and self-advocate [13]. While X has gained in popularity among young medical professionals, most noted through virtual communities like “#MedTwitter” and “#OrthoTwitter,” its utilization by medical students applying to Orthopedic Surgery residency is not well understood. This study aimed to characterize how the online orthopedic surgery collective uses “#OrthoTwitter” during Match Week and beyond.

Of the 370 tweets analyzed from 2021 to 2024, medical students authored almost half (47.7%), followed by residents and then fellows. Tweets authored by medical students also received the most likes, retweets, and replies, and most tweets were about a positive match result (39.3%). With that, there was a 2.2% increase in tweets from 2021 to 2024 and a significant increase in engagement with tweets about not matching. The number of tweets from DO physicians and osteopathic institutions increased significantly while the number of tweets from MD physicians and allopathic institutions decreased.

Since the COVID-19 pandemic, medical students, residents, program directors, and residency programs alike have taken to social media during Match Week [3,14]. In the first analysis of trends within the #OrthoTwitter community during Match Week, content posted by medical students received the most engagement over the past four years. Similar results were seen in social media patterns within general surgery; tweets with the most likes were medical student- and resident-centered [15]. Our study found that medical students received significantly more likes/tweets/replies than residents/fellows, PDs/APDs, residency programs, attendings, and third-party organizations. It is no surprise that Match Day, a celebration for medical students nationwide announcing their future specialties and institutions, garnered significant engagement on social media. This study confirmed that the number of celebratory tweets made up the majority of OrthoTwitter content during Match Week from 2021 to 2024. Holderread et al. found that 77% of orthopedic surgery residency social media accounts were created in 2020 in response to the COVID-19 pandemic and have only increased since [8]. With that, we can infer that increased medical student activity during Match Week is in response to the COVID-19 pandemic and the overall increased presence of residency programs on social media [16].

In contrast, there was a notable increase in engagement with tweets acknowledging not matching from 2021 to 2024. While social media has historically been a place for its users to post their successes and accomplishments, the data from our study challenges that [17]. Furthermore, the personal announcements of not matching were the second most engaged-with tweets during Match Week. It can be inferred that the #OrthoTwitter community can be a place for unmatched Ortho applicants to receive direction, mentorship, and alternate opportunities during Match Week [5,14]. With that, the presence of DO physicians, medical students, and osteopathic medical schools has increased by 17.5% over the last four years in comparison to MD and IMG professionals and institutions. DO students are often without home programs and have fewer resources and support. These students have found #OrthoTwitter to be a domain where they can have more access to opportunities, the ability to self-advocate, and market themselves on a large and public scale. This has the potential to garner the attention of residency programs, program directors, and other members of the #OrthoTwitter community [18].

The overall increase in #OrthoTwitter engagement in the last four years can be attributed to the COVID-19 pandemic and a collective shift in how medical students and residency programs disseminate and share information during a time when communication was limited [12,14]. More importantly, it seems that the use of social media in this way is here to stay. In a competitive field like orthopedic surgery with an increased number of unmatched applicants, the #OrthoTwitter community is a critical resource, especially for medical students who go unmatched and students without home programs [19,20]. Comparable findings were noted in the examination of the 2021-2022 Urology match, with a significant increase in advice for unmatched students described post-pandemic [9].

DO medical students and medical students without home programs who are interested in orthopedics should consider joining the #OrthoTwitter community for robust access to mentorship, exposure to residency programs, research and networking opportunities, as well as opportunities to self-advocate and self-promote [21].

Limitations

This study had several limitations that should be addressed. First, as this was a retrospective study, it is possible that not every post that included the #OrthoTwitter and #OrthoMatch hashtags was included in the study. Second, we only analyzed tweets containing the hashtags #OrthoTwitter and #OrthoMatch. Though most tweets utilized these hashtags during Match Week, it is likely that other associated tweets were not analyzed because they did not include these hashtags. Third, these posts were placed in categories such as “author type,” “institution association,” and “content type” by investigators. This is subjective, and some posts could have been placed in other categories. Fourth, although disagreements in classification were discussed and resolved as a group, we did not calculate a formal measure of inter-rater reliability. As a result, we could not quantify the level of agreement among reviewers before reaching consensus. Fifth, we did not adjust for multiple comparisons because each analysis examined a separate characteristic and addressed a different research question. However, given the exploratory and descriptive nature of these analyses, the reported p-values should be interpreted with appropriate caution. Lastly, because the data were manually collected, classified, and entered, the possibility of human error cannot be completely excluded, despite our review and reconciliation of the dataset and reported results.

## Conclusions

This study presents the first characterization of how medical students applying to orthopedic surgery utilize X during Match Week. The increase in X engagement between 2021 and 2024 demonstrates how the COVID-19 pandemic created a more robust and accessible community for orthopedic surgery-interested medical students and applicants to be a part of. Our study also illustrates that students who end up unmatched during Match Week seek advice and resources on #OrthoTwitter. Other findings include the increase in engagement and activity from DO medical students and professionals within the orthopedic surgery community. Students use #OrthoTwitter as an avenue to share their accomplishments, network, ask for advice, and seek new opportunities that they would not have known about otherwise. Future studies should include analyzing the use of #OrthoTwitter in unmatched orthopedic surgery applicants and students without home programs.
